# Sexual reactivation under aripiprazole in schizophrenia: a note for prescribers

**DOI:** 10.1139/jpn-25-0148

**Published:** 2025-10-24

**Authors:** Theo Korchia, Lena Palaniyappan

**Affiliations:** ^a^ Aix-Marseille University, Marseille, Provence-Alpes-Côte d'Azur, France; ^b^ Western University, London, Ontario, Canada

A 29-year-old man with schizophrenia, stable for over 3 years on risperidone 6 mg/day, presented with persistent negative symptoms, pronounced emotional blunting, and **complete sexual anhedonia**. He reported no libido, no arousal, and no sexual activity. Serum prolactin was elevated (72 ng/mL). Due to emotional flattening and metabolic side effects, risperidone was gradually tapered and **aripiprazole 10 mg/day** was initiated.

After 4 weeks, the patient spontaneously reported a **sudden return of sexual desire**, frequent morning erections, and resumed masturbation. This unexpected reactivation of sexual function, emerging rapidly after the switch, was initially experienced as intrusive and overwhelming, and caused him significant anxiety, as he described feeling “overstimulated” and unsure how to manage the intensity of his libido. Yet, from a clinical perspective, this phenomenon marked a turning point: a clear disruption of the previous emotional and motivational deadlock. Over the following 2 weeks, the sexual drive gradually stabilized, and he began to experience these changes as physiological and appropriate.

At week six, aripiprazole was increased to **15 mg/day** due to residual apathy and diminished emotional engagement. Interestingly, the improvement in sexual drive preceded and seemed to herald a broader affective reawakening. By week 10, the patient reported a **clear improvement in affective reactivity and relational capacity**. He had initiated a romantic relationship and described experiencing emotional and physical intimacy as both rewarding and manageable. Prolactin levels had normalized (17 ng/mL), and no adverse effects were observed.

This case illustrates a paradoxical but increasingly reported phenomenon: sexual reactivation under aripiprazole. While sexual dysfunction is widely accepted as a side effect of antipsychotics and present in more than half of patients with schizophrenia,^1^ its reemergence as hypersexuality often triggers clinical discomfort, uncertainty, or even pathologization—yet it may signify an early step in emotional or motivational recovery. In this case, the improvement likely reflects a combined effect of risperidone discontinuation with normalization of prolactin levels, together with the direct pharmacodynamic properties of aripiprazole. The relatively long half-life of aripiprazole may also contribute to the sustained course of these changes.

Aripiprazole's unique pharmacodynamic profile—partial agonism at D2 and 5-HT1A receptors, combined with 5-HT2A antagonism—may help explain its dual potential to both normalize sexual function^2^ and, in some cases, dysregulate it.^3^ Importantly, evidence suggests that following prior exposure to potent D2 antagonists, such as risperidone, compensatory receptor upregulation may enable aripiprazole to exert near-full agonist activity at D2 sites.^4^ This enhanced dopaminergic stimulation may underlie the abrupt emergence of hypersexuality or other impulse-related behaviors observed in some patients. Indeed, beyond sexual reactivation, aripiprazole has been associated with impulse control disorders, including pathological gambling, compulsive shopping, and binge eating.^5–7^ Regulatory authorities have also issued safety communications highlighting these risks,^8^^,^^9^ underscoring the need for clinicians to anticipate and contextualize such phenomena within the broader spectrum of dopaminergic reactivation. Unlike most antipsychotics, which exert strong dopaminergic blockade, aripiprazole exerts a stabilizing effect on dopaminergic tone, allowing for a partial restoration of activity in mesolimbic and mesocortical pathways*.* This modulation appears particularly relevant in patients with negative symptoms and antipsychotic-induced hyperprolactinemia, where dopaminergic hypoactivity underlies both motivational and sexual impairments.^10^ Clinically, aripiprazole exerts dose-dependent effects on sexual functioning: studies have shown that higher doses are associated with greater reductions in prolactin and improved sexual functioning when making a switch.^11^^,^^12^ From a pharmacodynamic perspective, however, it has been suggested that at sufficiently elevated doses, aripiprazole's partial agonist profile could approximate a more “neuroleptic-like” effect. While this may theoretically limit dopaminergic reactivation, the prolactin-sparing effects appear dose-dependent.^12^^,^^13^

In fact, many clinical trials and case series document improved sexual function following a switch from prolactin-elevating agents to aripiprazole,^14^ and current guidelines now recommend its use as a first-line option for treating antipsychotic-induced hyperprolactinemia.^15^ One pharmacodynamic analysis even suggests that aripiprazole's effects on sexual functioning and prolactin may be mediated by gene targets such as DRD2, HTR2A, MAO-B, and SLC6A3*,* pointing to a complex interaction between dopaminergic, serotonergic, and hormonal regulation.^16^

This case highlights how abrupt sexual reactivation under aripiprazole, though potentially disconcerting, may offer a valuable clinical signal—particularly in patients marked by negative symptoms and prolactin-related sexual suppression. Rather than viewing this resurgence as a side effect or behavioral excess, clinicians should consider its possible role as an early indicator of dopaminergic restitution and affective reconnection. Anticipating and framing these changes as part of the therapeutic trajectory can help reduce misinterpretation, foster engagement, and avoid premature diagnostic reconsideration. Close follow-up remains essential, not only to monitor for potential dyscontrol, but to assess how sexual and relational functioning evolve alongside broader clinical recovery. While the hypersexuality was transient and self-limited in this case, in some cases, such reactivation may persist and become troublesome, requiring dose adjustment or discontinuation; the optimal time frame before we can consider intervening in such cases remains unclear. Of note, a previous report describing improved libido following a similar switch from risperidone to aripiprazole highlights the inter-individual variations within a continuum of dopaminergic reactivation.^17^

More broadly, this case underscores the importance of integrating sexual health within psychiatric care—not as a secondary concern, but as a window into deeper neurobiological and psychosocial shifts that may herald meaningful improvement.
